# Supporting Scientific Advice through a Boundary Organization

**DOI:** 10.1002/gch2.201800018

**Published:** 2018-09-12

**Authors:** Eric B. Kennedy

**Affiliations:** ^1^ School of Administrative Studies York University Toronto Ontario M5T2B2 Canada

**Keywords:** boundary organizations, environmental policy, marine management, science advice, science policy

## Abstract

The complex socio‐environmental issues faced by society – including climate change, resource management, and fostering resiliency in landscapes that intermix human and natural features – are difficult challenges that demand contextually appropriate evidence‐based interventions. Institutional arrangements for providing scientific advice range from individual science advisors to large scientific committees or advisory councils, with a great deal of variation in their formal and informal structures. Regardless of the structuring of advisors, however, these arrangements face a common challenge: being required to speak to a wide range of issues in a time‐sensitive manner, each of which has extensive stakeholder communities, deep disciplinary knowledge, and many complicating attributes. It is argued that creating a formally associated, supporting boundary organization that is tasked with supporting the advisory functions can help to resolve this challenge and improve the overall quality of advice offered. Using a case study – the California Ocean Science Trust and its advice on coastal and ocean management issues – it is argued that boundary organizations can help science advisors maintain links with disparate stakeholder communities, adjudicate between competing forms of expertise, help to provide nuance in grappling with the tensions between science and politics, and support an “honest broker” advising function.

## 1 Introduction

For many, the ocean embodies serenity and peace. For those whose personal or professional lives intertwine with the coast, however, the ocean can all too often represent a set of fraught and contentious challenges. One such example took place in the mid‐2000s, when questions began to be raised in California about a controversial idea: whether components of oil and gas platforms should be left at sea when they were retired.

In many ways, this tension and controversy was predictable. At the end of their functional lives at sea, oil rigs eventually must be decommissioned. This is traditionally done through returning the platform to shore, which is a highly involved – and environmentally deleterious – process in nearly every phase from transport to disassembly. An alternative is to leave portions of the rig (particularly those below the surface) at sea, where they can potentially serve ecologically beneficial functions in supporting the development of reefs and fish refuges. While these purported benefits are valuable, the decision to leave significant components of a major human project at sea is challenging to protecting traditional norms of “nature” and “the pristine.”^1^

As such, the process of assessing the veracity of these claims (both at a local and general levels) was just as controversial as the actual debate over decommissioning. Those who could justify investing significant levels of financial support in this research – the industry – suffered from a credibility deficit precisely because of the incentives that enabled them to fund such research. Other communities who wished to support the research were similarly compromised. The sport fishing industry, for instance, stood to benefit from areas rich in fish and other biomass (the platform would serve as a magnet of sorts, drawing the prey to what could become dense fishing grounds). And, while other groups – such as university or government researchers – could conduct some of the research, they too relied on access and support from industries that questioned one another's credibility and operated on longer timescales than was conducive to the situation at hand. No group, therefore, could independently complete a study that would be seen as entirely neutral or objective by the others.

These are the kinds of situations in which boundary organizations thrive; spaces where science and values are intertwined, where conflicting interests risk inhibiting important collaborative work, and where relationships of trust and engagement must be forged across disciplinary and community lines. Boundary organizations (as introduced by Guston^2^) are so‐called for their roles in simultaneously spanning, reifying, being accountable to both sides of these gaps. Sometimes, they are university‐ or government‐based organizations (e.g., Parker and Crona^3^), while others are found in the private sector or elsewhere. They represent a specific way of institutionalizing not only mechanisms for science advice, but also institutional capacity to take into account social, economic, and political considerations. Although much of the research on boundary organizations has occurred within the North American and European context (including Guston's original case study of the Office of Technology Transfer), they have also been studied and applied around the globe, including in Vietnam,^4^ Brazil,^5^ and Indonesia,^6^ conducting work on both domestic and international issues.

These contested situations are also the spaces where scientific advice is highly sought. When decision‐makers are confronted with such challenges, they often espouse a desire for complete, objective, and dependable scientific advice that can guide (or justify) their decisions. Often, however, the science is hardly settled. The process of offering scientific advice, therefore, requires dealing with uncertainty, inconclusive results, multiple valid sets of data and interpretation, and contexts that may be well beyond what has been previously studied.^7^ Moreover, these scientific realities are overlaid on a sociopolitical landscape where different economic and political interests offer contrasting views of the ideal future.

In this paper, I argue that convening a boundary organization that is formally associated with the science advisor or advising body can help to augment the capacity of these more traditional approaches to scientific advice. Using the case of the California Ocean Science Trust, I illustrate the enhanced outcomes that can be achieved by coupling a boundary organization with science advising roles. I then consider at least four benefits that these organizations can bring to science advising: helping science advisors maintain links with disparate stakeholder communities, adjudicating between competing forms of expertise, supporting an “honest broker” advising function, and assisting in providing nuance in grappling with the tensions between science and politics.

## 2 Introducing Boundary Organizations

The challenges of environmental management described above likely sound familiar to those who have practiced in the space of science and policy writ large. These contexts are characterized by complex problems – those challenges that are highly intertwined, riddled with feedback loops, and nearly impossible to solve in a permanent fashion.^8^, ^9^ Moreover, these are contested spaces where the differing values and vested interests of various stakeholders make resolution difficult. Finally, the existing institutional infrastructures are often poorly matched to the nature of the problems, which typically span many traditional departments and agencies.

The concept of “boundary organizations” offers one institutional model for approaching these fraught problems. Boundary organizations are so‐named for the roles that they can play in simultaneously crossing and reifying the boundaries between different sectors, agencies, stakeholders, and paradigms (e.g., science vs policy or stakeholders vs industry, among others). In their strongest form, boundary organizations have the power to mediate and facilitate effective relationships between viewpoints, institutions, and communities that have historically been in conflict.^2^, ^10^

Guston defined boundary organizations as those institutions that “internalize the contingent character of the science/politics boundary” through an ongoing process of negotiating and stabilizing the division between the two worlds.^10^ While this is broadly compatible with functions of science advice, it does not necessitate that boundary organizations serve any sort of advisory role. They could, for instance, serve instead (either exclusively or concurrently) in translation, coordination, or trust‐building functions between a wide variety of stakeholder groups, for instance. Instead, for Guston, boundary organizations are characterized by three defining attributes:

1) they provide a space that legitimizes the creation and use of boundary objects and standardized packages;
2) they involve the participation of both principals and agents, as well as specialized (or professionalized) mediators; and
3) they exist on the frontier of two relatively distinct social worlds with definite lines of responsibility and accountability to each.

Academics and practitioners alike have largely embraced the concept. For academics, boundary organizations offer an intuitive, common sense concept – a simple name for a regularly encountered phenomenon – that is malleable enough to be applied to a variety of case studies (e.g., by Bateman^11^). For practitioners, the label often offers a way of articulating the interdisciplinary, difficult‐to‐describe work that they do in such facilitative spaces. Since Guston's initial theorizing of the concept and a subsequent (in 2001) expansion of the theory, over 2400 citations have been made to his introductions, and double that number of papers have made mention of the theory. Subsequent definitions have cast a broader theoretical role than Guston first laid out via his criteria when he focused on the simultaneous reifying and spanning of boundaries. Some, for instance, have defined boundary organizations as “mediating between science and policy and facilitating the interaction between actors on either side or who cross the boundary” (Cash).^12^ Still others have opened the definition even further, considering organizations for their ability to foster collaboration, preserve competing stakeholders' interests, and enrol actors from opposing sides (e.g., O'Mahony and Bechky^13^). Again, in these broader interpretations, boundary organizations sit at the interface between science and society, but do not automatically or always function in a formal advisory role – they can just as easily serve to facilitate knowledge exchange without taking advisory positions, for instance, or to provide an institutional foundation for the co‐production of new knowledge by multiple actors.

The three formal criteria provided within Guston's definition, however, are indicative of prospective attributes of boundary organizations that suggest their value within the realm of science advising. The use of boundary objects and standardized packages, for instance, often translates into hosting workshops or dialogues, or developing written reports and syntheses on the state of a contemporary issue. The involvement criterion demands stakeholder relationships that are grounded in trust, understanding, and a willingness to engage. And, the explicit differentiation of “distinct” social worlds serves to justify their prolonged existence: the boundary organization serves to translate, mediate, and ultimately bridge between these two worlds. In short, boundary organizations are equipped to serve on the interfaces of complex, multistakeholder environmental, technoscientific, and societal challenges. As an example of these theories in action – and the ways in which a boundary organization can be built to support pre‐existing or novel science advising functions – I next consider the case of the California Ocean Science Trust.

## 3 Case Study: The California Ocean Science Trust

The example of oil and gas platform decommissioning (see ref. 14 for further details on this case in particular) is only one of many situations that have demanded a combination of science advice and boundary organization functions over the past decades. After Alaska and Florida, California has the third longest coastline in the USA, at well over 800 miles.^15^ In 2000, the government of California passed the “California Resources Stewardship Act” (CORSA). The legislation was motivated by several factors, including environmental protection, public benefit, and the estimated $53 billion dollars that the coastline brought to California in industrial, recreational, port, and naval activities.^16^ The bill provided authorization for the Secretary of the Resources Agency to establish the Ocean Science Trust and to fund it to “encourage coordinated, multiagency, and multi‐institution approaches to ocean resource science” (CORSA^16^ p. 1).

The legislation framed the problem of marine governance as a threefold challenge. First, despite “making progress in ocean management efforts,” (p. 2) California faced ongoing challenges such as species protection, pollution prevention, and coastal erosion that were being inadequately addressed through existing government efforts. Second, the legislation argued that state and federal agencies “often lack[ed] basic information on which to base decisions,” compounded by difficulties in coordinating efforts across agency boundaries (p. 2). Finally, the legislation pointed to significant investments already being made in research endeavors along the coast that could be better mobilized to eliminate information gaps, effort duplication, missed opportunities, and unusable information (p. 2).

From the onset of the act, therefore, the challenge of marine governance was framed as a coupled effort. One part was the need for effective scientific advice, linking existing work with evidence‐based decisions to protect the economic and environmental value of the coastline. Yet, because this stemmed from problems of research coordination and agency misalignment, these outcomes could not be achieved through advising alone. As such, CORSA made provision for a trust to be established that would fulfill these organizing and coordinating functions in a way that a single science advisor could not.

### 3.1 Situating Ocean Science Trust (OST) in Context: The Network of Marine Governance in California

The California Ocean Trust enabled by CORSA as a nonprofit 501(c)(3) public benefit corporation (now named the “California Ocean Science Trust” or OST) was only one part of a much larger institutional landscape on marine governance that arose through the early 2000s in California. In fact, the particular role that OST would play in the marine governance regime in California was not well defined to either OST or lawmakers. It was not until 2004, when then‐California governor Arnold Schwarzenegger signed into law the “California Ocean Protection Act” (COPA), that OST's role in marine governance in California became much clearer in law and practice alike.

The major thrust of COPA was to establish the Ocean Protection Council (OPC). The council was made up of five key actors: the Secretary of the Natural Resources Agency, the Secretary of Environmental Protection, and the Chair of the State Lands Commission, alongside two members of the public appointed by the Governor for their qualifications and “knowledge of, interest in, and experience in the protection and conservation of coastal waters and ocean ecosystems” (Public Resources Code Section 35600–35625). These members are responsible for several tasks, including coordinating the various marine‐related activities of several agencies; establishing policies on data collection, evaluation, and sharing; recommending policy changes to the state Governor and Legislature; and establishing a “Science Advisory Team” (OPC‐SAT) of

“…distinguished scientists to assist the council… At the request of the council, the science advisory team may convene to identify, develop, and prioritize subjects and questions for research or investigation, and review and evaluate results of research or investigations to provide information for the council's activities.” (Public Resources Code Section 35600–35625)

**Figure 1:**
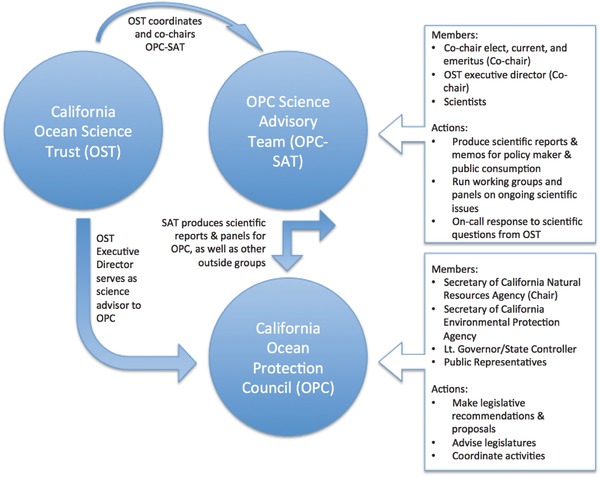
Simplified representation of organizational relationships between OST, OPC, and the OPC‐SAT.

In both the legislation (3516.a.4) and in practice, the OST became the secretariat of the Science Advisory Team (OPC‐SAT). Moreover, given the large mandate of the OPC relative to its council and staff support, OST began to take on significant roles in supporting and advising the OPC through original research, synthesis and translation of scientific findings, and maintaining networks and relationships with the marine scientific community in California. Finally, the Executive Director of OST was assigned to serve as the science advisor to the OPC (which includes three high‐ranking government officials and has an official mandate to provide policy advice to the Governor and Legislature). A simplified series of these relationships are illustrated in **Figure** **1** (below), which indicates the ways in which OST serves as a direct science advisor to the OPC, and an indirect science advisor to the Californian government.

### 3.2 The Ocean Science Trust as a Boundary Organization

To consider how OST serves as a boundary organization, I conducted a case study investigation of the organization using mixed methods. I began by analyzing public facing documents, reports, and legislation produced by and informing OST, using these documents providing general context about their work and approaches. From this material, I developed a semistructured interview protocol for both OST members and stakeholders who had experience interacting with the organization (including both policy‐makers and academics). I conducted 28 interviews over a two‐year period, using these temporally stratified interviews to track the development of projects over time. I conducted two visits to the organization in Oakland, California, using these observational visits to provide further context about projects, methods, and informal perspectives. Because of the small size of the organization, quotations that follow from staff members and stakeholders are kept anonymous.

In examining the OST and its role as a boundary organization, recall the three criteria as defined by Guston.^10^ The first is that a boundary organization “legitimizes the creation and use of boundary objects and standardized packages.” For OST, the boundary objects are typically found in the form of technical assessment reports produced on particular areas of science need identified by the OPC or other state partners. The framing of these documents (which can range from relatively short reports through to multi‐year, six‐ and seven‐figure assessment projects) as technical assessments is quite intentional. In conversation with OST staff, several described the importance of assessments that focused on scientific evidence rather than making policy judgments, in order to maintain OST's status as a neutral facilitator with whom all stakeholders could engage. Moreover, the process by which these reports are created is highly iterative: scientists, community members, and industry are generally invited to be part of a process that convenes disparate interests through a conversation about developing and editing these reports. By situating itself as the creator of technical reports and, more broadly, as convener of the OPC‐SAT, OST legitimated its role as a boundary organization through creating boundary objects that government, stakeholders, and scientists could engage with while debating complex environmental challenges.

Guston's second criterion requires that boundary organizations must include principals, agents, and specialized mediators. In many cases, the principals for OST are connected to public goods laid out by CORSA and COPA: the interests of citizens, the environment, and other public values.^17^ The agents consist of a multitude of government officials, both in formal capacities (e.g., as members of the OPC or government scientists participating in research) and informal roles (e.g., in signaling an area for future research, or participating as a stakeholder in a consultation process). Most importantly, OST staff themselves serve as the mediators, addressing the concerns of the agents while being accountable through their formative legislation to the citizenry writ large.

The OST also meets the third criteria; which is sitting on the “frontier” of distinct worlds. In this case, the primary relationship is much like that faced by science advisors: between scientists and the policy community. For OST, this is mediated by serving as science advisor to the OPC – influence, therefore, is not through directly advising a first minister or the executive branch, but rather through the agency heads that comprise the Ocean Protection Council and the many departments that sit within those agencies.

It is worth noting that OST also serves the simultaneous functions of spanning and reifying the distinction between science and politics. Although boundary organizations bridge this gap, they also engage in actions of demarcation in separating the expertise, evidence, and experience required in each. When one OST staff member, for instance, described the importance of “making sure we were only reviewing the science, not the management or policy,” they tacitly engaged with three arguments: a) drawing a distinction between the realms of science and management/policy; b) indicating that it was indeed even possible to disentangle the two (that science and politics are distinct and different worlds; contra the argument that these worlds are indeed always blurred, muddled, and bleeding into each other (see ref. 18 as this argument relates to boundary organizations)); and c) setting up the role that OST could play in bridging that gap. In context, this distinction seemed largely appropriate (OST had been asked to review specific scientific information on questions scoped and defined by policy institutions). Yet, it indicates the variable role OST plays on different projects, ranging from facilitating problem definition through to answered predefined technical questions.

With respect to providing science advice, OST takes on a multilayered role. The most explicit science advising occurs through the appointment of the executive director as science advisor to the Ocean Protection Council. This can range from long‐term or ongoing monitoring projects through to timely questions asked on marine‐related issues at OPC meetings. But, OST also offers a significant volume of science advice through other channels, including the production of reports directly consumed by other agencies or departments, as well as responses to time‐sensitive questions. Finally, OST takes on a series of other functions often associated with science advisors, including networking the scientific community, serving as a conduit toward specific experts, and providing knowledge resources to the larger management and practice communities.

## 4 Supporting Science Advice through a Boundary Organization

While the urge to link science and decision‐making is broadly agreed upon as important, actually forging these linkages requires significant effort and capacity alike. The issues on which science‐informed policy may be most important – problems like climate change, environmental management, and social welfare – are also those challenges that are highly complex^9^ or “superwicked.”^19^ These problems involve large number of stakeholder groups (including industry, state and local governments, and communities), competing expertise, scientific uncertainty, and divergent values – as well as a very wide range of different topics. Accordingly, it is incredibly difficult for a small advisory committee – let alone a single advisor – to have not only the range of disciplinary expertise to credibly advise on all these issues. By contrast, a boundary organization can serve as a supporting structure, by assembling a broader range of both disciplinary and metacognitive expertise in order to support high quality scientific advice. In this section, I offer four short examples of how this can be achieved. These are not meant to be an exhaustive summary, but rather to indicate a partial picture of the advantages offered by developing a boundary organization to support science advising.

### 4.1 Maintaining Links with Disparate Stakeholder Communities

One emblematic challenge of science advising is the large number of stakeholder communities involved in decision‐making. This includes not only a large number of scientists who produce relevant research, but also an array of government agencies, industries, communities, and special interest groups. This problem is multiplied when science advisors need to speak to a wide range of subjects, be it marine and coastal management, or even science, technology, and innovation in general. At its simplest level, a boundary organization provides additional staff and resource capacity for forming, maintaining, and drawing on these stakeholder partnerships – a larger team, with an explicit mission of developing and maintaining relationships, can sustain a much larger network that can then be called upon during relevant moments by the science advisor themselves.

For OST, the boundary organization offers this supplemental capacity. The OST director's ability to maintain relationships with stakeholders is augmented by a wider team within the organization, each of whom can focus on particular issue areas and networks of relevant parties. Moreover, the disciplinary variation within OST staff assists with this capacity: some OST staffers have deep disciplinary training in marine science and are able to leverage that credibility to develop relationships with government, academic, and industry scientists. By contrast, other members of the staff have experience in areas like public engagement or the social studies of science, providing competencies for working with more diverse stakeholder groups.

### 4.2 Adjudicating between Competing Forms of Expertise

In addition to sheer time and capacity, boundary organizations can offer an intermediate layer between the science advisor(s) and stakeholders who offer competing claims to expertise. In the cast of OST, the boundary organization can identify the relevant experts needed to offer advice on a given topic, facilitate their participation, and create boundary objects (such as synthesis reports) that could communicate these many different kinds of expert knowledge in a way relevant to the science advisor. Like many boundary organizations, therefore, an important form of expertise with OST is facilitation – expertise in convening effective meetings, mediating contentious discussions, and anticipating logistical, ideological, and personal roadblocks.

Negotiating expertise, however, is not always simply additive or deferential (e.g., according expert status to all and presenting all ideas as identical kinds of inputs). In many cases, as suggested by Guston^10^ boundary organizations can play an important demarcating role between different forms of expertise. At OST, for instance, this meant cultivating internal expertise in procedural, philosophical, and reflective assessment to support drawing distinctions between the technical/scientific and nontechnical forms of expert input. Researchers at OST described the willingness to define these lines as important to their commitment to producing objective, credible, and science‐driven assessments. Moreover, these distinctions percolated down into the very framing of projects – the very notion, for instance, that scientific expertise could be neatly separated from both internal and external values (see ref. 20).

While most consideration of expertise focused on the content and specialization of the experts, OST was also acutely aware of the way that perceptions of expertise affected how much traction their advice might be able to achieve and how to translate expertise in a way that would actually be useful to science advice processes. When discussing how a particular project should be positioned, for instance, one researcher argued that “this will only work… if the thought leadership comes from the SAT.” This statement meant more than simply the need to include experts from the SAT; rather, it was meant to articulate that despite the relevant expertise existing in many different places, the project would be taken more seriously, more credibly, more powerfully if decision‐makers associated it with a body reputed for its expertise. Like with almost all functions of boundary work and science advising, researchers at OST were concerned not only that the quality of the work be near perfect, but also that the outsider perceptions of the quality of the work were similarly high.

### 4.3 Grappling with the Tensions between Science and Politics

Negotiating the appropriate role for expertise also requires understanding that a) simply inserting more science into decision‐making processes is not always the ideal solution, and b) scientific insights need to be seen in their larger political and value‐based contexts. Even if there was broad agreement on the nature of the problem or the ideal solution, the data necessary to chart a path forward may not exist, thanks to a misalignment between the research being conducted and decision‐maker needs^21^, ^22^ or the intractability of the problem itself. Injecting more science into controversial topics can in fact often make their resolution more difficult, as it is usually not “a lack of scientific understanding but… the lack of coherence among competing scientific understandings” that leads to unresolved controversies (Sarewitz^7^), as well as propagating an inaccurate “deficit model” of science (see ref. 23; see also refs. 24 and 25). This is acknowledged by voices within the science advice community. Science advice is important as one of “a wide range of inputs,” for instance, that government decision‐makers must consult (alongside stakeholder input, public values, other considerations) to be “confident that a rigorous and objective assessment of all available information was made” (CSTAS^26^ pp. 2–3).

Boundary organizations provide a structure for acknowledging that more needs to be done than simply “drop off” science at a proverbial decision‐making loading dock.^27^ In OST, for instance, this manifests in researchers who understand the importance of having “absolutely objective analysis,” while also “maximizing the chance science will get used” by creating “the pathway for science to flow in, in ways that are credible.” This means paying attention to the social, structural, and political realities in order to use science to “set that agenda, and set up scientists to be impactful.” At the same time, boundary organization personnel were attuned to issues of the uncertainty of scientific knowledge, the social and political values that influenced the issues at hand, and the need to offer advice with imperfect and incomplete scientific data (the “best currently available evidence,” as it was termed).

As Stirling emphasizes, science advice also must support “more plural and conditional methods” that “illuminate a variety of alternative reasonable interpretations” that are consistent with available evidence.^28^ Likewise, advisors may be wise to foreground uncertainty by developing particular analytical and communication tools, like spread analyses or uncertainty matrices.^29^ As a boundary organization, OST also created capacity for developing guidance about how uncertainty could be disclosed and discussed in productive and transparent ways.^30^

### 4.4 Supporting an “Honest Broker” Approach to Science Advising

A highly influential text on science advising is Roger Pielke Jr's “The Honest Broker: Making Sense of Science in Policy and Politics.”^31^ After sketching out the difficulty of providing science advice in polarized contexts (which Pielke Jr terms “abortion politics” which, unlike “tornado politics,” lacks agreed upon problem definitions and courses of action), Pielke Jr offers a series of possible roles scientists and their backers can embody. Of particular interest to science advisors is the “Honest Broker of Policy Alternatives,” a scientist or advisor who seeks to clarify existing policy options and offer new, broadened options for going forward. Although there are important critiques of the political theory underpinning Piekle Jr's argument,^32^ the honest broker role offers an idealized and aspirational vision of what a science advisor can do. Honest brokers must carefully navigate translational, curatorial, and distributional roles in knowledge management to connect stakeholders with usable knowledge in transparent and nonpartisan ways.

Given their role as a boundary organization, OST, the researchers at OST described similar ambitions in supporting the provisioning of science advice. One described, for instance, how OST functioned as “an opportunity expert, not a content expert,” wherein the organization's raison d'être was broadening the questions, evidence, and alternatives available to decision‐makers. Moreover, there was a sense of pride in the behind‐the‐scenes work OST did to offer policy makers and community members with a series of information and alternatives that they could take, grapple with, and own. “Sometimes,” one researcher explained, “the success of the effort often comes at the expense of us being a visible actor.”

The case of OST illustrates an interesting aspect of science advisors' and boundary organizations' interactions with honest broker theory. Like with questions of expertise, for OST, being perceived as an honest broker was just as important as being an honest broker. This is not to suggest that OST was trying to advance a particular agenda. Indeed, in interviews with internal researchers and external clients, there was not any sentiment but a well‐earned reputation for serving the public good, and a desire to earnestly present policy makers with the highest quality science possible. The line between Pielke Jr's honest broker and his “stealth issue advocate,” however, is a particularly fine one, particularly when considering the powerful ways in which an honest broker could (intentionally or unintentionally) frame a problem; the values underlying its definition and research; and the breadth of policy alternatives available.

## 5 Conclusion

Society increasingly must grapple with complex and multistakeholder socio‐environmental problems. These collective challenges necessitate both scientific advice and the mediation of often contested stakeholder relations. In this paper, I have explored the way that boundary organizations can productively contribute to these challenges, and particularly the ways in which they can help support science advisors or advisory structures. I began by focusing on the four criteria laid out by Guston (the creation of boundary objects; the principal, agent, and mediator roles; and the accountability to distinct social worlds) to explore the tangible attributes of boundary organizations in real‐world practice. I then provided a case study example of one such organization – the Ocean Science Trust – that has been designed specifically to assist in the provisioning of timely, relevant, and high‐quality science advice.

In turn, this example has allowed for an analysis of four different ways in which boundary organizations can provide additional capacity, resources, and insights in the service of science advising: developing strong links with stakeholder communities, capacity for adjudicating between competing forms of expertise, assistance in grappling with the tensions between science and politics, and supporting an “honest broker” approach to science advice. These are functions of significant utility, as they can serve to complement science advisors or advisory committees that may lack internal capacity to focus on these functions. These are not simply the result of increased logistical roles or added staffing. Rather, as I have illustrated through examining the structure and practice of OST, boundary organizations provide a distinctive institution for producing, mobilizing, and translating knowledge.

While creating such a boundary organization in support of a science advisor undoubtedly has a cost – requiring staffing and resourcing – it provides significant additional capacity to support efforts at science advice. Moreover, there is reason to believe that it has multiplicative value: the diversity of expertise, perspectives, and experiences within such an organization – as well as the emphasis on procedural work and metacognitive skills – offers something different than simply a larger committee of science advisors. While not the only way of supporting a science advisor or advisory team, boundary organizations may offer a unique way of enhancing the impact, reliability, and quality of science advice.

## Conflict of Interest

The authors declare no conflict of interest.
